# Maximizing the Treatment Benefit of tDCS in Neurodegenerative Anomia

**DOI:** 10.3389/fnins.2019.01231

**Published:** 2019-11-22

**Authors:** Carlos Roncero, Erik Service, Marco De Caro, Aleksandar Popov, Alexander Thiel, Stephan Probst, Howard Chertkow

**Affiliations:** ^1^Rotman Research Institute, Baycrest Health Sciences, Toronto, ON, Canada; ^2^Lady Davis Institute for Medical Research, Jewish General Hospital, Montreal, QC, Canada; ^3^Department of Nuclear Medicine, McGill University, Montreal, QC, Canada; ^4^Department of Medicine (Neurology), University of Toronto, Toronto, ON, Canada

**Keywords:** tDCS, anomia, object naming, PPA, training

## Abstract

We evaluated whether transcranial direct current stimulation (tDCS) in two different montages could improve picture naming abilities in participants with anomic Alzheimer Disease or Frontotemporal dementia.

**Methods:**

Utilizing a double-blind cross-over design, twelve participants were trained on picture naming over a series of 10 sessions with 30 min of anodal (2 mA) tDCS stimulation to either the left inferior parietotemporal region (P3), the left dorsolateral prefrontal cortex (F3), or sham stimulation. We evaluated performance on a trained picture naming list, an equivalent novel untrained list, and additional neuropsychological tasks.

**Results:**

For trained item picture naming, significantly larger improvement was seen for real stimulation vs. sham stimulation for both the DLPFC and left inferior parieto-temporal stimulation montages at the end of the stimulation sessions. The parieto-temporal montage remained superior to sham 2 weeks poststimulation. Significant improvement vs. sham was also seen for novel “untrained” item picture naming 2 weeks post-stimulation when the parieto-temporal montage was given, whereas no change was observed when the DLPFC montage was given. Finally, comparing groups when they received the parieto-temporal montage, participants with semantic variant Primary Progressive Aphasia (PPA) showed the least improvement for untrained items after their sessions. Scores on the additional neuropsychological tasks were unchanged.

**Conclusion:**

tDCS stimulation has promise as a treatment for individuals with anomia arising from neurodegenerative disease, but its effectiveness can vary depending on the training given, the montage location used, as well as a participants’ diagnosis.

## Introduction

There has been increasing interest in the past 20 year in the potential for electrical stimulation to positively benefit brain function, both in response to advances in the field, as well as the lack of pharmaceutical treatments for brain-related impairments (Rosa and Lisanby, 2012). This excitement may have been sparked by two landmark papers which found direct current applied to the human scalp could produce excitability changes lasting beyond the time of stimulation (Priori et al., 1998; Nitsche and Paulus, 2000, 2001). The realization that electrical stimulation can produce long-lasting symptom reduction beyond the time of administration in turn made it realistic as a practical form of therapy for brain disease states. This potential of electrical stimulation as a symptomatic treatment, in the present context where there is a lack of drug treatments for neurological conditions, has driven stimulation studies to increasingly include individuals who either fail to respond to a particular medication or for whom no medicated treatment exists. In other words, electrical stimulation is often examined as the non-drug alternative (Guleyupoglu et al., 2013); representing a potential treatment for conditions where there are few if any effective therapies. Modern techniques such as tDCS are nearly painless because they deliver a low level of current, making them highly acceptable. For these reasons, electrical stimulation has become increasingly popular as a treatment plan for brain ailments that lack an obvious therapy.

One of the major adopters of this emerging treatment plan was clinicians examining stroke patients, who often enrolled their patients in physical therapy programs designed to improve symptoms. These pioneering researchers examined if training results would be enhanced when combined with transcranial direct current stimulation (tDCS), which was a marked contrast from the historical application of electrical stimulation that had typically been given under sedation or at rest. Numerous studies reported stronger training effects due to tDCS (for a review, see Marquez et al., 2015). For example, language training for post-stroke aphasia was found to be enhanced when combined with tDCS (Floel et al., 2008; Schlaug et al., 2008; Baker et al., 2010; Holland and Crinion, 2012). Due to these positive findings, the vast majority of tDCS studies now involve *functional targeting*: enhancing the effects of a particular training program by combining it with tDCS. In turn, the number of potential tDCS studies is limited only by the number of training programs that can be imagined, and has led to an exponential number of published tDCS articles that incorporate a wide range of topics; including studies with healthy adults for behaviors unrelated to a particular illness (e.g., meditation; Badran et al., 2017). The recent creation of computer modeling software (Bikson et al., 2012) also allows researchers to now include *anatomical targeting* in their studies: determining the best electrode configuration that will drive stimulation toward a targeted neural area. Consequently, studies are often designed to incorporate both functional and anatomical targeting; for example, stroke patients may complete a training program while stroke-related brain areas are stimulated, or healthy adults might meditate while stimulating brain areas believed to underlie meditation. The combination of functional and anatomical targeting is expected to produce an improvement surpassing training or stimulation alone.

In this paper, we will focus on studies examining the use of tDCS for Primary Progressive Aphasia (PPA), which lacks designated drug therapies. tDCS has become a popular form of electrical stimulation; possibly because it is relatively easy to administer. For the vast majority of tDCS studies, two electrodes (anode and cathode) are placed within sponges that have been pre-soaked in saline and secured to the scalp using a head-strap. Both electrodes are connected to a power source that allows for a direct current to pass through the brain from electrode to electrode for a duration and intensity level pre-defined by the researcher. As done for post-stroke aphasia, tDCS studies have examined if a particular form of speech-language therapy, the principal current treatment for PPA, would have a greater and longer lasting benefit when combined with tDCS. To our knowledge, nine studies have been completed, with more than half in the last 2 years, and all demonstrated that training was more effective when combined with tDCS; despite administering different protocols. To visualize this variability, key aspects of each study are noted in Table 1.

**TABLE 1:** Past tDCS studies with primary progressive aphasia (PPA) participants.

**Study**	**Participants**	**Training**	**Anode**	**Cathode**	**Intensity**	**Duration**	**Sessions**	**Outcome**
Wang et al. (2013)	One non-fluent PPA	Refused training	Morning: Left posterior perisylvian region Afternoon: Left broca’s area	Extracephalic: Right shoulder	1.2 mA	20 min	5 sham 5 tDCS	Auditory word-picture identification, picture naming, oral word reading, and word repetition all improved after administering tDCS; decline 2 months post-stimulation
Cotelli et al. (2014)	16 Agrammatic PPA	Speech therapy: repetition of target word, articulatory suppression, picture naming, reading words	Left dorsolateral prefrontal cortex	Extracephalic: Right arm	2 mA	25 min	10 sessions of either sham or tDCS	Improvement for treated items was greater for anodal tDCS than sham tDCS, for untreated items, both montages had similar levels of improvement
Tsapkini et al. (2014)	Two non-fluent PPA, four logopenic PPA	Participants wrote a letter or letter combination corresponding to a given phoneme	Left inferior frontal gyrus	Extracephalic: Right cheek	2 mA	20 min	15 sessions each condition (tDCS and sham)	Improved Spelling; improvements greater and lasted longer (2 months) in real condition, untrained items improved in real condition only
Gervits et al. (2016)	Two non-fluent PPA, four logopenic PPA	Narrating wordless children’s picture books	Left frontotemporal region	Left occipitoparietal region	1.5 mA	20 min	10 sessions of tDCS	Improvements in speech production and grammatical correctness lasting 3 months; no sham condition
Teichmann et al. (2016)	12 semantic PPA	Simple visuomotor task: Press a button when a particular object reaches the edge of the screen. Done to maintain vigilance.	Session 1: Left temporal (FT7 to FT9) Session 2: Left fronto-orbital	Session 1: Right frontoorbital Session 2: Right temporal (FT8 to FT10)	1.59 mA	20 min	3 sessions (one session per condition)	Compared to sham, tDCS sessions improved responses to questions written in a verbal format on a semantic matching task, reaction times to questions regarding living items were faster after anode fronto-orbital and cathode right temporal stimulation.
Roncero et al. (2017)	Six non-fluent PPA, two logopenic PPA, two semantic PPA	Repeated naming of items incorrectly named at that day’s session	Left inferior parietotemporal region	Right frontoorbital region	2 mA	30 min	10 sessions each Condition (tDCS and sham)	Picture naming scores for trained and untrained items improved more in the real condition; lasting at least 2 weeks
Hung et al. (2017)	One logopenic PPA, three semantic PPA, one early onset AD	Repeated spontaneous naming, sentence production, and semantic feature generation	Left temporoparietal region	Centered over the forehead	1.5 mA	20 min	10 sessions of tDCS	Improved naming for trained items lasting 6 months; there was no sham condition. Zero improvement for untrained items. No change observed for early onset AD participant.
McConathey et al. (2017)	Six non-fluent PPA, one logopenic PPA	Narrating wordless children’s picture books	Left frontotemporal region	Left occipitoparietal region	1.5 mA	20 min	10 sessions of either sham or tDCS	Individuals who scored low at baseline had greater propensity to improve when given real tDCS relative to sham tDCS
Tsapkini et al. (2018)	14 non-fluent PPA, 12 logopenic PPA, 10 semantic PPA	Confrontation verbal and written naming; errors corrected and repeated	Left inferior frontal gyrus	Extracephalic: Right cheek	2 mA	20 min	15 sessions each condition (tDCS and sham)	Written naming letter accuracy for trained and untrained items improved for logopenic and non-fluent PPA, no change found for semantic PPA

It can be observed in Table 1 that the various studies are similar for certain variables: all had an intensity level greater than 1.0 mA, but no greater than 2 mA; stimulation in all studies lasted at least 20 min, but no more than 30; and all studies had the intended goal of combining training with stimulation.

Because these studies all reported positive results, these aspects of the paradigm seem crucial for success. Furthermore, these results were found despite typically having a sample size smaller than 10, which suggests the effect may be quite robust. More variable across the studies was the exact training program used, the type of participants included, and the relative position of the anode and cathode electrodes. Regarding the training program, the results reflect functional targeting: the evaluated behavior was also the behavior trained. For example, spelling training led to improved spelling (Tsapkini et al., 2014, 2018), while naming training led to improved naming (Cotelli et al., 2014; Roncero et al., 2017), and open-ended narration led to improved speech production and grammatical correctness (Gervits et al., 2016). Perhaps more surprising, improvement repeatedly occurred despite the variant positioning of the anode and cathode electrodes. For example, Cotelli et al. (2014) placed the anode electrode over the frontal cortices, while Roncero et al. (2017) placed it over the inferior parietal lobe (IPL), yet both report improved naming. Studies have also typically reported results for a mixed group of PPA participants rather than a single sub-type; thus, it is unclear if the results found were true for all PPA sub-types or if the positive effects found were driven by a particular sub-type within the group examined. For example, Tsapkini et al. (2014) initially reported an improvement for spelling when giving tDCS with spelling training to PPA participants, but in a more recent study, where PPA sub-types were examined and compared (Tsapkini et al., 2018), it was found that the treatment was effective for people with non-fluent PPA, and those with logopenic PPA, but perhaps ineffective for people with semantic PPA.

In summary, while studies appear to have coalesced around certain paradigm heuristics (intensity, duration, combining stimulation with targeted training), other variables remain less decided like the relative position of the anode and cathode electrodes, and whether tDCS is more effective for certain forms of PPA. Clearly, any potential treatment requires precision by further understanding when that effect can be present or absent, and under what conditions. In this manner, future clinicians will be better positioned to recommend when and to whom tDCS treatment should be given. However, aside from Tsapkini et al. (2018) who compared PPA sub-types, no other study to date has directly compared the effectiveness of different tDCS variables for PPA, including which montage may be best for a particular training program. Considering the array of stimulation sites displayed in Table 1, one may even radically ask whether the site of stimulation is an important variable? For example, while Roncero et al. (2017) had demonstrated that tDCS applied over the left inferior parietal lobule (IPL) improved naming ability in people with PPA, one can ask whether a different montage [e.g., anode over the dorsolateral prefrontal cortex (DLPFC)] would have produced the same results. Therefore, we examined the importance of electrode positioning in this study by directly comparing the results of three montages for the same group of PPA participants: (1) A temporal-parietal montage, where the anode electrode was placed over the inferior left parietal-posterior left temporal lobe region and the cathode electrode placed over the right supraorbital region; (2) A sham stimulation montage with the same arrangement; and (3) A DLPFC Montage, where the anode electrode was placed over the left DLPFC and cathode electrode over the right deltoid muscle. This paradigm allows for a direct comparison of the montages by examining the results of each montage for the same training program and group of participants.

Both the DLPFC and parietal-temporal lobe are considered important parts of a complex cortical network associated with language processing; albeit with different presumed roles. Whereas the DLPFC is activated during executive function processes, the temporal parietal region is more involved in semantic control processes, in particular when naming objects (Binder and Desai, 2011). Thus, stimulation to the temporal parietal region may produce more improvement for naming than stimulation to the DLPFC. However, it is also possible that stimulation to either area will produce similar results because both areas are part of the same cortical network. Stimulation to either area would produce increased activation of the network as a whole, with the distinct anode locations simply representing different activation entry-points. In this case, results for the different montages would be similar because both montages similarly stimulate the same neural network.

A secondary goal, in light of the results found by Tsapkini et al. (2018), was checking if the results found for all participants would be similar for the different PPA sub-types. Because PPA sub-types are affected by distinct areas of atrophy, stimulating areas that correspond best or closest to these areas of atrophy may be critical for efficacy. For example, logopenic PPA individuals, who have deficits primarily related to word retrieval, have atrophy largely affecting the parietal lobe; thus, stimulation may be most beneficial when applied to the parietaltemporal area. In contrast, people with non-fluent PPA, whose deficits primarily involve issues with articulation, have atrophy primarily in the frontal cortices and may benefit more when stimulation is applied to the DLPFC rather than the parietal lobe. Finally, semantic PPA individuals, who exhibit semantic memory deficits in addition to word finding difficulties, have atrophy localized at the anterior temporal lobe (ATL) and may fail to benefit from either montage in the current study as neither the DLPFC montage nor the parietal-temporal montage are predicted to directly to stimulate the ATL. Consistent with this argument, Tsapkini et al. (2018) found a lack of tDCS benefit for semantic PPA individuals, compared to other PPA sub-types, when tDCS was administrated with the anode electrode over the inferior frontal gyrus and the cathode over the right cheek.

In summary, we tested the hypothesis that training with tDCS was no better than training alone for improving naming function in people with PPA. Based on past results, we predicted training with tDCS would be superior to the results found when training is paired with SHAM stimulation. At the same time, we will compare real rounds of tDCS to examine if the results are different depending on the montage used. For example, because the temporal-parietal areas are important for semantic control functions, stronger results may be found when the anode electrode is placed over this area. Finally, we will examine if the results are different depending on PPA type. For example, Tsapkini et al. (2018) found results were weaker for participant living with svPPA.

## Materials and Methods

### Participants

All participants were patients already given a formal diagnosis of PPA at the Memory Clinic of the Jewish General Hospital in Montreal, QC, Canada after having undergone both neurological scans (e.g., FDG PET, MRI) and neuropsychological testing. Informed consent was also obtained at the Jewish General Hospital, whose internal research board approved this study. Inclusion criteria included fluency in either English or French, absence of non-degenerative neurological disorders (e.g., stroke), as well as observable anomia: defined as scoring below a cut-off point for normal performance on the spontaneous naming task of the Cambridge Semantic Battery (Adlam et al., 2010). This battery is commonly used to detect anomia and associated semantic deficits in people living with dementia. There were no medical exclusions, but participants were asked to refrain as far as possible from altering their medications during the study and report if any medication change occurred during the course of the study. Individuals with pace-makers or any neurologically implanted device (e.g., shunt) were excluded. Finally, only participants scoring above 10 on the mini-mental status exam (Folstein et al., 1975) were recruited.

### Study Design

The study was designed to have participants attend three rounds of stimulation, each consisting of a different montage (parietal-temporal, DLPFC, SHAM). Because our primary goal was a direct comparison of different montages, the order of the montages was counter-balanced across participants using stratified randomization to ensure an equal number of participants per montage order. Each round consisted of a baseline evaluation, and 10 stimulation sessions. These 10 stimulation session took place over the course of 3 weeks (Week 1: baseline evaluation on Tuesday, stimulation sessions on Thursday and Friday; Week 2: stimulation sessions on Monday, Tuesday, Thursday, Friday; Week 3: stimulation sessions on Monday, Tuesday, Wednesday, final stimulation and evaluation session on Thursday). In the baseline evaluation, two picture lists were presented (one to be trained, one to be left untrained) and participants were asked to name the pictures presented. In the subsequent nine stimulation sessions, the participants would receive stimulation while engaged in a training protocol to improve naming accuracy. More specifically, at the beginning of each training session, the participant would be asked to name the items from the designated trained list concurrently with the commencement of the tDCS machine. For any items named incorrectly, the participant would be asked to repeatedly name that item over the course of the training session (see section “Language Training Protocol” for details). In the final and tenth stimulation session of each round, training was replaced with a second evaluation where the two picture lists from the baseline evaluation, the list that was trained and the list left untrained, were again presented to check if the participant could now name more items on each list. Two weeks after this final stimulation session, as well as 2 months later, participants were given the same evaluation, but without the administration of tDCS. Each round was roughly 2 months apart; thus, participants needed 6 months to complete the study.

In summary, evaluation took place before the first stimulation session, at the final stimulation session that started with 30 min of real or sham tDCS, as well as 2 weeks and 2 months later without stimulation. The subsequent round started no sooner than 2 months later, at which point participants returned to receive an alternate type of stimulation, but otherwise underwent the same procedure as done in the previous round.

### tDCS Methods

Prior to the baseline evaluation, all participants underwent a structural MRI to approximate the location of the target brain areas vis- à-vis their scalp with the aid of a Transcranial magnetic stimulation neural-navigation device (Magstim Rapid stimulator, double 70-mm coil, United Kingdom). These target brain areas were then marked with a pen and identified as the location where the electrodes should be placed for a particular montage. To ensure researchers would be able to reproduce this dot in subsequent rounds, measurements and co-ordinates from CZ were noted and recorded to map-out the location on the person’s scalp. Subsequently, for all montages, electrodes measuring 5 by 7 cm were placed vertically on the participants’ scalp in the designated area with the dot roughly in the center of the electrode. The electrodes would sit on the scalp, within sponges that had been pre-soaked using syringes. Approximately 25 ml of saline (0.09%) was applied to each sponge just before the electrode was slipped within and subsequently placed on the participants’ scalp. Rubber straps were used to secure the sponges in place.

**FIGURE 1:**
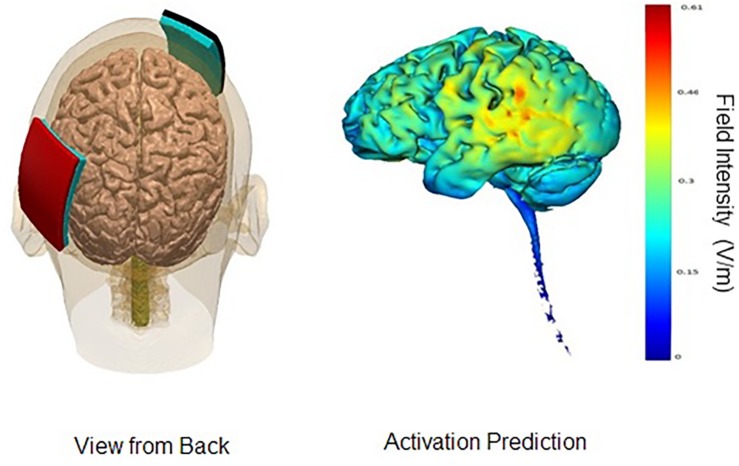
Parieto-Temporal Montage. Images display the parieto-temporal tDCS montage with anode on left parieto-temporal area and cathode on right supraorbital lobe. Activation prediction based on modeling software from Soterix HD-Explore; color gradients reflect increasing levels of predicted intensity from blue to red, where red reflects a peak intensity of 0.61 mA. Images courtesy of Soterix.

**FIGURE 2:**
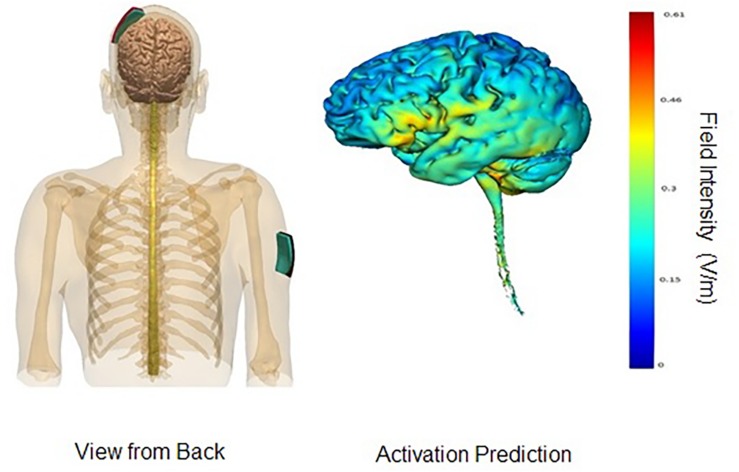
DLPFC montage. Images display the DLPFC tDCS montage with anode on left DLPFC area and cathode on right deltoid muscle. Activation prediction based on modeling software from Soterix HD-Explore; color gradients reflect increasing levels of predicted intensity from blue to red, where red reflects a peak intensity of 0.61 mA. Images courtesy of Soterix.

In the parieto-temporal montage, the sponge covered an area that included Brodmann’s areas 39 and 40, as well as the superior sections of Brodmann’s areas 21, 22, 37, 41, and 42. In other words, the sponge was placed diagonally north-east from the person’s left ear, approximately TP9. The reference electrode (cathode) was placed on the right fronto-orbital area; the bottom edge of the sponge brought down to the person’s right eyebrow. For the DLPFC montage, the anode electrode would be placed with the center over F3, and the cathode electrode over the right deltoid muscle. An elastic armband was used to keep the sponge in place; rather than using metal clips, the end of the armband was tucked under itself. Figures 1, 2 display the montages used, as well the peak areas of activation as predicted by computer modeling software (HD-EXPLORE, Soterix). HD-Explore is a tDCS current flow simulation software provided by Soterix Medical. The software was introduced to make individualized modeling accessible to clinical users in order to make dose decisions (number of electrodes to use, what electrode placement to use, etc.). The modeling methods employed in HDExplore are based on extensive prior work (Datta et al., 2012). These methods have been subsequently validated using *in vivo* intracranial recordings in humans (Huang et al., 2017). As done in our previous study where positive results were observed, a CE-certified DC-Stimulator MC (NeuroConn GmbH, Ilmenau, Germany) would be used to administer tDCS for 30 min at 2 mA. To ensure impedance levels would be below 5.0 kΩ for all participants, where side-effects are perceived substantially less, syringes would be used to apply further solution to the area underneath the sponges.

### Blinding

At the low levels of impedance used in the present study (2 mA), participants quickly habituate to the feeling of stimulation. Indeed, stimulation is generally most perceived at the beginning of a session when the charge ramps up (i.e., is increasing), or at the end of the session when the charge ramps down (i.e., fades to extinction; Siebner et al., 2004). It is effectively these changes in intensity that are perceived by participants. For this reason, sham stimulation, as done in the current study, is administered after an initial sixty-second ramp-up that is perceived well by participants, but then ceases, to mimic the habituation effect experienced during real stimulation. To mimic the final phase where the charge ramps down, the machine produces its own ramp-down at the end of the session despite having administered no current during the session. By mimicking the ramp-up and ramp-down of real tDCS stimulation, sham stimulation was expected to successfully blind participants of their designated stimulation condition (i.e., real or sham; Gandiga et al., 2006).

In the present study, participants and evaluators were blinded; however, trainers were aware if the stimulation being administered was real or sham. As our participants are individuals who suffer from cognitive impairment, it is possible that they will spontaneously behave in a manner that requires attention. Thus, trainers ensured the standard study protocol was administered and completed by the participants correctly, but also ensured our participant’s safety and well-being during the sessions. For this reason, all of the trainers in the study were individuals who had received certified training from the Alzheimer’s Society of Montreal regarding how to interact and help individuals with cognitive decline. However, to further ensure the correct response was always initiated, it was also important that the trainer be unblinded to the condition and have an advanced understanding of the tDCS machine and its functions. Some of our participants are often disinhibited and therefore do not always have control over their impulses; leading them to involuntarily try to scratch under the sponge and lift or shift their head montage regardless of condition as they often complain about the wetness of the sponge or the straps which are present across all conditions. Such situations, during a real stimulation session requires an intervention, in terms of re-adjustment or complete reset of the montage itself, in order to ensure good contact quality is maintained. Also, many of our participants are more anxious than average, even during sham sessions, and may report discomfort related to the intensity of the stimulation, the straps used, the wetness of the sponges, or something completely unrelated to tDCS. In such cases, the condition (sham or real) can dictate the correct course of action. In a sham condition, one would more likely investigate the tightness, maybe address the anxiety first, whereas in the case of real stimulation, the trainer might redirect our attention to the machine, the positioning of the wires, the contact quality, or the desire to add additional saline.

Indeed, it can be argued that even if a person were sitting beside the participant monitoring the tDCS machine, the trainer would be able to deduce if the session was real or sham. Unlike the participants, the trainer works and observes several different participants in a day, and can therefore notice subtle differences between participants that could indicate if stimulation was sham or real. For example, they may notice that the redness from the sponges is stronger in certain participants compared to others, and begin to believe those participants with redder marks are those receiving real stimulation. In contrast to trainers, evaluators in this study only witnessed stimulation at evaluation 2, and in addition to being blind of stimulation condition, were left largely ignorant regarding the tDCS machine and set-up. They also entered the room only when the machine was ready to be started after an initial successful test ramp-up, and the tDCS set-up was maintained on the participant’s head throughout the entire session, even after stimulation had diminished, to avoid revealing any aspects such as redness that could indicate for the evaluator if the session was real or sham. These evaluations with stimulation also only occurred roughly once a month, in contrast to trainers who observe tDCS on a daily basis, across different participants, throughout the entire month. Therefore, the opportunity to witness differences in behavior related to the type of tDCS stimulation given is greatly diminished as they more commonly interact with the participants without any tDCS set-up. In summary, this was a double-blind study because both subjects and evaluators were completely blind as to whether participants received real or sham tDCS.

### Naming Stimuli

The naming stimuli were taken from the Snodgrass and Vanderwart (1980) image set. The images were normed by 20 elderly normal controls (mean age 72 years), who were asked to name the image and provide a familiarity rating from one (not at all familiar) to 7 (very familiar). We then eliminated any images where the name provided was inconsistent across participants, was incorrectly named by more than two participants, or had a familiarity rating lower than 3.0. The remaining images were subsequently organized into their semantic categories, ranked for familiarity, and divided into three 60-item lists hereafter called Naming 1, 2, and 3. In this manner, each list was equally familiar and had a similar number of exemplars from each semantic category. As previously discussed, one list of items was used for daily training sessions in each round of stimulation, which coincided with an initial 30 min of tDCS stimulation, while another naming list was left untrained. More specifically, in the first round of the experiment, Naming 1 was trained, and Naming 2 was left untrained, whereas for round two, Naming 2 was trained and Naming 3 was left untrained. In Round 3, Naming 3 was trained, while Naming 1 was used again to assess the effect for untrained items. This paradigm allowed us to assess changes in naming pre and post tDCS for both “trained” and “untrained” picture items.

### Secondary Tasks

An additional set of general cognition tasks were also included to assess participant’s performance in additional domains, and to check if the parieto-temporal or DLPFC tDCS montages would lead to higher scores compared to sham. No training for these tasks was ever carried out, which included: forward and backward digit span from the WAIS-IV (Wechsler, 2008) verbal fluency (F, A, S, Animals), and two assessments of general cognition: the Montreal Cognitive Assessment (MoCA; Nasreddine et al., 2005) and the Mini-Mental State Examination (MMSE; Folstein et al., 1975).

### Language Training Protocol

Each training session had four phases: (1) tDCS set-up; (2) initially presenting the items from the designated trained list; (3) producing a list of items missed in the initial presentation; (4) training missed items. Set-up was completed by a research assistant at the beginning of each training session to ensure the first 30 min of the training session were concurrent with the administration of tDCS. For the initial presentation of items, the participant was presented the images individually on a 15.6” Dell laptop using the computer program P*resentation*^®^ (Version 18.0, Neurobehavioral Systems, Inc., Berkeley, CA, United States)^1^, a stimulus delivery and experiment control software for neuroscience. Each image had a display size of 900 × 900 pixels.

Following a fixation cross, each image on the designated trained naming list was presented for 6 s. During this period, the participant was given the chance to name the item on the screen. No cues nor any kind of feedback was provided by the trainers during this part of the session. The trainer then produced a list of items to be trained by noting which items were incorrectly named by the participant. These missed images were then ranked in familiarity based on established norms from the most familiar to the least familiar, and the five most familiar missed items formed the first five-item study group. The trainer then presented each item of the study group one by one to the participant, naming each item in front of the participant; this was repeated two more times. The training of missed items then consisted of presenting the images of the study group separately on individual sheets of paper, this time asking the participant to again name each item. Whenever an item was presented, the trainer would note whether each item was now correctly named by the participant. When participants had difficulty remembering the name of the item, the trainer would give phonological cues (starts with a.) or semantic cues (In Halloween, you carve a.). These cues often helped the participant, but successful naming after a cue was still considered a miss. The items from this initial study group were presented in another display cycle, one-by-one, each time noting whether the participant could correctly name the item, followed by another display cycle of the items to the participant. Items named correctly each cycle, three cycles in a row, were then removed from the initial five-item study group and replaced by additional items from the list of items missed during the initial presentation. For example, if all five items had been correctly named three times, then they were replaced with the next five most familiar missed items, but if only two items were correctly named three times across the cycles, then the items named successfully less than three times were kept, while the two successfully named items were replaced by the next two most familiar items that were missed during the initial presentation. The trainer then presented this group of five items, again noting each display cycle if the item was named correctly. Items presented previously still required successful naming by the participant three cycles in a row for elimination from the study group. After three cycles, the trainer would again note the number of items named correctly three display cycles in a row and replace those items with the next most familiar missed items that had yet to be trainedto create the next study group. The training session continued in this manner until all missed items were trained. However, to avoid exhaustion, sessions were never longer than 2 h, even if some missed items were left untrained that session. Although this form of training for naming has never been formally ratified, it did produce naming improvement in our previous study and was expected to improve naming ability again in the current study.

### Statistical Analyses

In the current study design, participants were their own controls. Thus, we ran repeated-measures Anovas with stimulation (DLPFC, parieto-temporal, Sham) and time (Pre-Stimulation, Final Stimulation Session, 2 Week Follow-up, and 2-Month Follow-up) as within-subject factors for both trained and untrained items to examine how the real montages compared to each other and if they produced more improvement than sham stimulation. Toward this goal, and assuming a significant time by stimulation interaction, trend analyses would be subsequently run to determine if scores in the different montages increased, remained the same, or decreased, which would then be followed by comparisons to baseline if the trend was significant. Having the same participants undergo all three montages facilitates recruitment and reduces inter-participant variability.

### Carry-Over and Disease Progression Effects

The present study design is complicated by potential carry-over effects, whereby participants after completing a round of real tDCS stimulation may maintain that improvement rather than having a wash-out effect during the 2-month gap between stimulation rounds. In the opposite direction, inevitable disease progression in some participants may be severe enough to make certain participants untestable despite initially being compatible with the study. To check for both carry-over and disease progression effects, we included montage order as a between-subject factor in our repeated measures Anovas. In addition, we also checked for carry-over effects at the individual level by calculating the mean and standard deviation of each participant’s baselines scores for trained and untrained items and omitting any participants with a baseline score that was more than 1.5 standard deviations from their own baseline mean. In other words, the baseline scores for the naming lists at the beginning of each round were examined to check to what degree these scores fluctuated from one another. More generally, if the scores obtained in round 2 at baseline for a participant were substantially higher than those obtained in round 1. In round 3, baseline scores were again compared to check if the round 3 baselines scores were substantially higher than those observed in rounds 2 and 1 for that participant. Substantially higher or lower was defined as a score greater or lower than 1.5 standard deviations from the mean of baseline scores. The disease progression effect was handled by the fact that participants who experienced observable declines were removed from the study and their data was omitted from analyses. Thus, all presented data comes from participants who demonstrated stable cognitive function throughout the study.

## Results

### Participants

**FIGURE 3:**
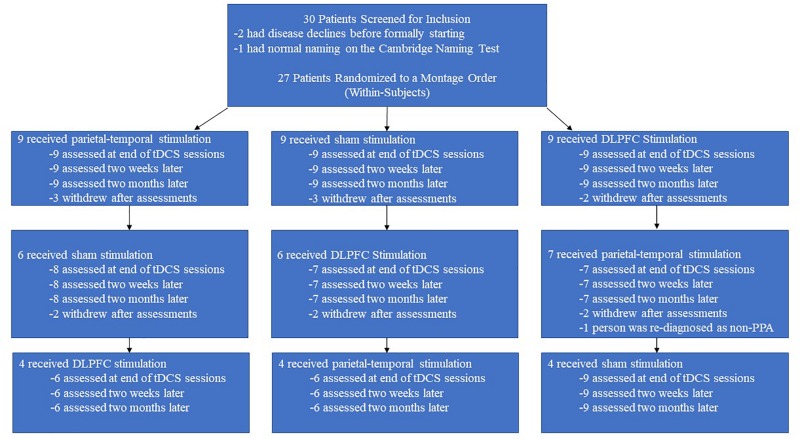
Attrition rate over time. The above flowchart displays the attrition rate for the separate counter-balance groups across the different rounds. Each counter-balance group began with an initial cohort of nine participants, but attrition led to only 4 people completing the three rounds without disease progression effects.

We screened 30 individuals and ultimately enrolled 27 individuals with PPA: 10 with nfPPA, 10 with logoPPA, and 7 with sPPA. In the initial screening, it was also verified that participants could attempt to name an image when prompted to ensure all participants were testable. However, the length of the study (6 months) and the number of training and evaluation sessions requested each round (13) led to a high level of attrition as outlined in Figure 3. Four participants withdrew from the study after the first round, and six participants withdrew after the second round. Furthermore, four participants had severe disease progression over the course of the study to the point where they were unable to follow the study’s training protocol. Finally, one participant completed the study, but their diagnosis was changed from PPA to Motor Neuron Disease; thus, this person’s data was also omitted. Ultimately, the data from 12 participants was available and useable for analyses, whose demographic data is presented in Table 2. The data from these 12 participants were also checked for carry-over effects. For these participants, the baselines scores across rounds were relatively similar and never exceeded 1.5 standard deviations. Thus, for the presented data, we conclude that any improvements observed for naming ability were temporary before returning to baseline. The 12 participants also subdivided into three PPA groups of equal size (4 nfPPA, 4 logoPPA, 4svPPA).

**TABLE 2:** Patient diagnostic and general cognition data.

**Patient**	**Diagnosis,**	**Age**	**Sex**	**Education**	**MoCA**	**MMSE**	**C. Naming**
**code**	**PPA type**		**(M/F)**	**(years)**			**Score**
NF1	FTD, nf PPA	72	M	15	24	28	57
NF2	FTD, nf PPA	62	M	11	13	17	59
NF3	FTD, nf PPA	75	M	11	21	26	58
NF4	FTD, nf PPA	72	F	11	11	17	44
LG1	AD, logo PPA	64	M	18	7	12	44
LG2	AD, logo PPA	61	F	8	9	18	28
LG3	AD, logo PPA	63	F	18	24	27	56
LG4	AD, logo PPA	69	M	11	10	17	30
SV1	FTD, sv PPA	54	M	16	16	28	8
SV2	FTD, sv PPA	59	M	14	19	25	26
SV3	FTD, sv PPA	71	F	18	22	26	9
SV4	FTD, sv PPA	63	M	11	17	18	15

*FTD, frontotemporal dementia; AD, Alzheimer’s Disease; PPA, primary progressive aphasia. PPA sub-types: nf PPA, non-fluent PPA; logo PPA, logopenic PPA; sv, semantic variant PPA; C. Naming is the spontaneous naming score obtained on the naming task from the cambridge semantic battery (max = 64, Normal elderly control M = 62, s.d. = 2).*

### Effects of tDCS Compared to Sham

Data was analyzed from the 12 participants who neither withdrew, had a change of diagnosis, nor had a severe progression decline. For both trained and untrained items, we ran repeated-measures anovas with stimulation (DLPFC, parieto-temporal, Sham) and time (Pre-Stimulation, Final Stimulation Session, 2-Week Follow-up, and 2-Month Follow-up) as within-subject factors, and montage order as a between-subject factor.

The main effect of stimulation was significant for trained items [*F*(2,18) = 6.82, *p* < 0.01] and marginally significant for untrained items [*F*(2,18) = 2.80, *p* = 0.09], while the main effect of time was significant for trained items [*F*(3,27) = 17.11, *p* < 0.001] and marginally significant for untrained items [*F*(3,27) = 2.52, *p* = 0.08]. Crucially, the critical statistic of interest, the interaction between stimulation and time, was significant for both trained [*F*(6,54) = 2.80, *p* < 0.05] and untrained [*F*(6,54) = 2.50, *p* < 0.05] items, while the stimulation by time by order statistic was non-significant for both trained [*F*(12,54) = 1.57, *n.s.*] and untrained items [*F*(12,54) = 0.99, *n.s.*]. Thus, the type of stimulation received had an impact on the performance by participants over time and was true for all montage orders.

Having found a significant interaction, we next ran a trend analysis for each montage condition to better understand how the changes in each montage were different over time. More specifically, these analyses would allow us to check if performance for a particular montage had increased over time (a positively linear trend), decreased over time (a negatively linear trend), or if performance had initially increased, but then declined over time (a quadratic trend). Results indicated there was a significant quadratic trend for both trained items [*F*(1,11) = 14.20, *p* < 0.01] and untrained items [*F*(1,11) = 8.08, *p* < 0.05] when the parietal-temporal montage was given; thus, there had been an increase followed by a decrease for both trained and untrained items. When the DLPFC montage was given, there was also a significant quadratic trend for trained items [*F*(1,11) = 21.80, *p* < 0.01], but no significant trend was observed for untrained items; thus, there was an increase followed by a decrease for trained items, but no change noted for untrained items. was noted when the DLPFC montage was given. Finally, when sham stimulation was given, a quadratic trend was again observed for trained items [*F*(1,11) = 28.57, *p* < 0.001], but a significant downward linear trend for untrained items [*F*(1,11) = 2.50, *p* < 0.05]; thus, even for sham, there was a temporary increase followed by a decrease, but the scores for untrained items became progressively worse over time.

### Comparisons to Baseline

**FIGURE 4:**
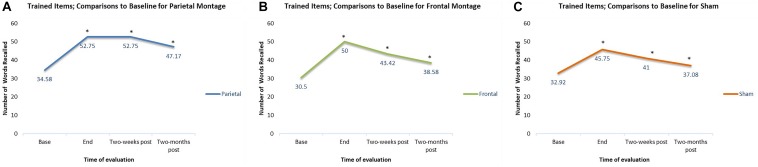
Comparisons to baseline for the naming scores of trained items at end of stimulation sessions, 2-weeks post-stimulation, and 2-months post-stimulation. ^∗^ significant at *p* < 0.05 for that evaluation compared to the scores obtained at baseline. **(A–C)** Displays the results for the different montages. **(A)** Parieto-temporal montage, **(B)** DLPFC montage, and **(C)** SHAM.

In addition to checking for the general trends of each individual montage, we also checked at what time interval the results for each montage were significantly different from baseline. For trained items, compared to baseline, participants had significantly higher scores at the end of stimulation sessions regardless the montage given (parieto-temporal, *t*(11) = 4.18, *p* < 0.01; DLPFC, *t*(11) = 5.41, *p* < 0.001; Sham, *t*(11) = 4.83, *p* < 0.01). Two weeks post-stimulation, scores remained significantly higher than baselines for all montage (parietotemporal, *t*(11) = 4.79, *p* < 0.01; DLPFC *t*(11) = 5.48, *p* < 0.001, Sham, *t*(11) = 4.22, *p* < 0.01). Finally, scores for trained items were significantly higher than baseline even when examined 2-months post-stimulation (parieto-temporal *t*(11) = 3.79, *p* < 0.01; DLPFC *t*(11) = 4.80, *p* < 0.01, Sham, *t*(11) = 2.76, *p* < 0.05). Thus, for all three montages, scores at the final tDCS session, as well as 2 week and 2 months post-stimulation, were all significantly greater than baseline. These comparisons are displayed in Figure 4.

**FIGURE 5:**
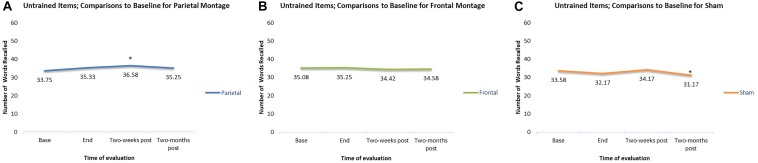
Comparisons to baseline for the naming scores of untrained items at end of stimulation sessions, 2-weeks poststimulation, and 2-months post-stimulation. ^∗^ significant at *p* < 0.05 for that evaluation compared to the scores obtained at baseline. **(A–C)** Displays the results for the different montages. **(A)** Parieto-temporal montage, **(B)** DLPFC montage, and **(C)** SHAM.

In contrast, for untrained items, there were only two significant differences when scores were compared to baseline. First, when participants received the parieto-temporal montage, their scores were significantly greater than baseline when examined 2-weeks post-stimulation. Second, when scores in the sham montage condition were compared to baseline, scores 2-months post stimulation were actually significantly worse than those obtained at baseline. Meanwhile, no change from baselines were observed for participants during the DLPFC montage condition. These comparisons are displayed in Figure 5.

### Comparison of Montages at Each Evaluation

**FIGURE 6:**
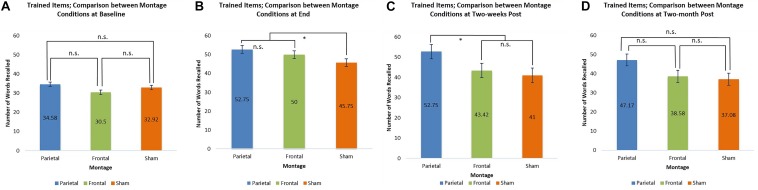
Comparison of montage conditions for trained items at each evaluation: Baseline, End of Stimulation Sessions, 2-Weeks Post Stimulation, 2-Months Post Stimulation. ^∗^ significant at *p* < 0.05, meaning the scores for one montage were higher than those obtained for another montage at that evaluation. **(A–D)** Refer to the different times of evaluation. **(A)** baseline evaluation, **(B)** evaluation at final tDCS session, **(C)** evaluation two-weeks post-stimulation, and **(D)** two-months post-stimulation.

At baseline, the scores for trained items were similar for all three montages. At the end of the stimulation sessions, however, scores for the parieto-temporal montage and the DLPFC montage were significantly higher than those obtained when stimulation was sham (parietal-temporal vs. sham *t*(11) = 2.92, *p* < 0.05, DLPFC vs. sham, *t*(11) = 2.38, *p* < 0.05). This result was again found 2 weeks post-stimulation for participants when they received parieto-temporal stimulation as the scores for this montage at this evaluation were higher than those achieved by the same participants when they received sham stimulation (parietaltemporal vs. sham *t*(11) = 2.47, *p* < 0.05). In contrast, the scores achieved by participants receiving DLPFC stimulation were lower 2 weeks post-stimulation and found to be similar to those achieved by participants when they received sham stimulation. Thus, only parietal-temporal stimulation had scores higher than sham stimulation 2 weeks post-stimulation. However, at 2 months post-stimulation, participants’ scores when they received the parietal-temporal montage became similar to those found in the other montage conditions. Thus, montages had similar scores at baseline, and 2 months post-stimulation, but the real tDCS conditions produced larger scores at the end of the stimulation sessions, as well as 2 weeks post stimulation for the parieto-temporal montage. These comparisons are presented below in Figure 6.

**FIGURE 7:**
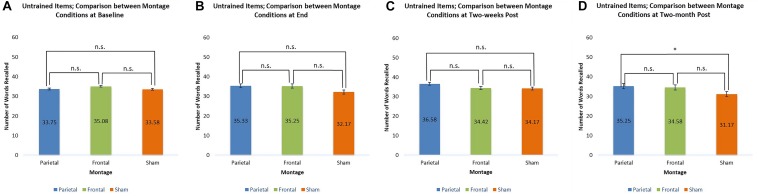
Comparison of montage conditions for untrained items at each evaluation: Baseline, End of Stimulation Sessions, 2 Weeks Post Stimulation, 2-Months Post Stimulation. ^∗^ significant at *p* < 0.05, meaning the scores for one montage were higher than those obtained for another montage at that evaluation. **(A–D)** Refer to the different times of evaluation. **(A)** baseline evaluation, **(B)** evaluation at final tDCS session, **(C)** evaluation two-weeks post-stimulation, and **(D)** two-months post-stimulation.

For untrained items, only one significant comparison emerged. Two-months post-stimulation, it was found that scores achieved by participants receiving the parieto-temporal montage were significantly larger than those achieved by participants when they received sham stimulation [*t*(11) = 2.60, *p* < 0.05]. These comparisons are presented below in Figure 7.

### Participant Type

**FIGURE 8:**
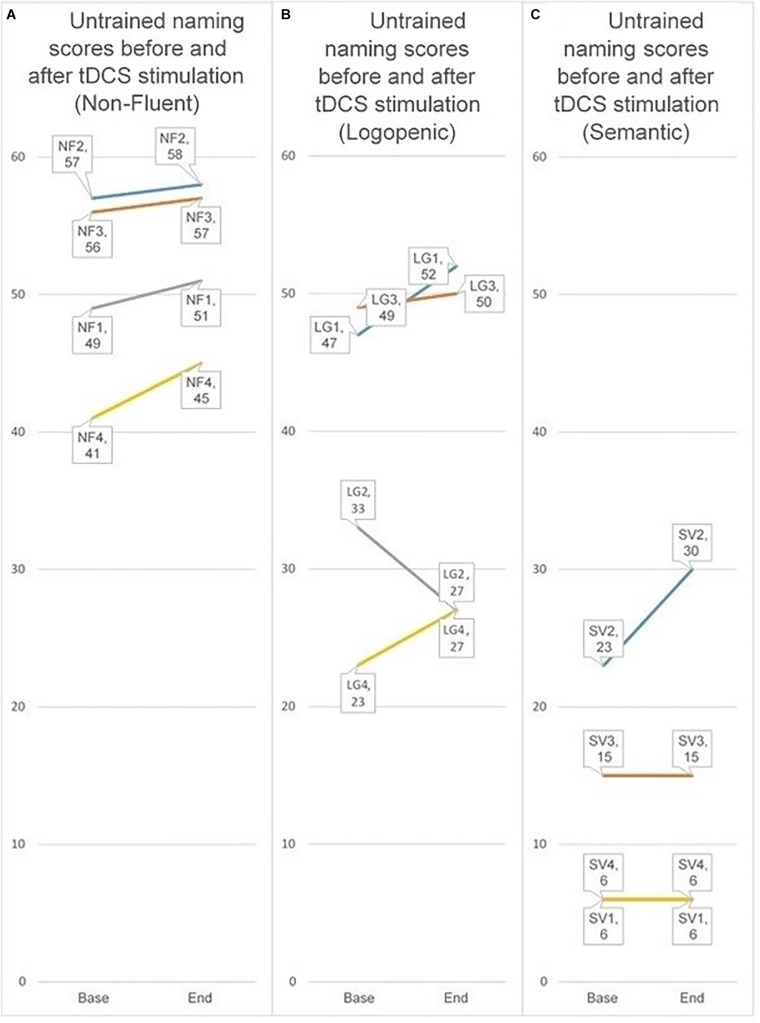
Individual scores for untrained items when receiving the parieto-temporal montage at baseline and at the final stimulation session. The three groups represent the three PPA sub-types (nfPPA, logoPPA, svPPA). Lines between the score at baseline and the score at the first subsequent evaluation are used to help visualize if a person’s score increased, decreased, or stayed the same. **(A–C)** Refer to the different PPA sub-types. **(A)** nfPPA, **(B)** logoPPA, and **(C)** svPPA.

Because our participant groups were small, we have chosen to simply present the data rather than conduct formal analyses. For this reason, results shown should be read cautiously. We present the individual groups by displaying for each participant in that group whether he or she improved their baseline score for untrained items at the final stimulation session when the parieto-temporal montage condition. We chose this montage because participants on a whole showed a significant improvement for untrained items only in the parieto-temporal condition. Furthermore, we compared the baseline score to the score in the final stimulation session because it best reflects the effect of stimulation received without any additional time periods. Comparing the score in the final tDCS session to baseline, all four non-fluent FTD participants showed an improvement, as did three of four logopenic AD participants. In contrast, only one participant with semantic variant FTD improved for untrained items. The contrasts are displayed below in Figure 8.

### Secondary Task Results

As done for trained and untrained items, we carried out repeated-measures Anovas for all participants on the other tasks administered during evaluation [MoCA, MMSE, Verbal Fluency, Digit Span (forward and backward)]. None of these comparisons were significant.

### Adverse Effects and Debriefing

Because impedance levels were always brought to a level where current sensations are minimally felt, participants were able to tolerate well the stimulation received. The debriefing sessions served as our informal assessment for whether blinding was successful. In debriefing sessions, participants reported being unable to distinguish which rounds involved sham stimulation. In contrast, many reported they found the sensation similar across rounds and some reported forgetting that one of the rounds would be sham stimulation. Therefore, 0/12 participants reported being able to tell whether a particular round involved real stimulation or sham.

## Discussion

In this proof of principal study, we compared the effects of training with real or sham tDCS to improve naming for a set of trained and untrained items. In general, tDCS produced an improvement in picture naming for a mixed group of PPA participants that was superior to training and sham for both trained and untrained items. Crucially, to our knowledge, this is the first study that compared two tDCS montages in addition to sham for the same group of participants. Because of this study design, we can discuss how the different montages appear to have different advantages for improving anomia when combined with language training. At the end of the stimulation sessions, real tDCS produced larger improvements than sham both for items that were trained and items left untrained regardless the montage. However, differences related to the montage used were also observed with increasing time. More specifically, although both the DLPC and parietal-temporal montages lead to greater improvement for trained items when participants were evaluated at the final stimulation session, only the parietal-temporal montage maintained this advantage over sham stimulation when participants were evaluated 2 weeks after their final stimulation session. The parietaltemporal montage was also the only montage where a significant improvement was found for untrained items 2 weeks post-stimulation. In contrast, no change was observed for untrained items in the DLPFC montage condition, while sham stimulation lead to a significant decrease 2 months post-stimulation. These different results may reflect the functions of the respective areas. The key role of the DLPFC for working memory processes are likely highly used during training, and tDCS stimulation to this area could have produce stronger effects related to training. Thus, we observe the largest improvements for trained items when DLPFC stimulation is given. Stimulation to this area, however, may have improved general working memory processes rather than processes specific to naming; the outcome measure in this study. The inferior parietaltemporal region, for example, is known as a key brain area for language control, especially naming (Whitney et al., 2012). In turn, it is possible that stimulating key areas related to naming rather than areas related working memory lead to longer-lasting and more generalized improvement.

The relevant position of the cathode electrode also deserves attention (Bikson et al., 2010).

Comparing the modeling patterns in Figures 1, 2, it can be observed that stimulation generalizes to more brain areas in the parieto-temporal montage (Figure 2). These differences are likely related to the placement of the relevant cathode electrode and the current flow between the two electrodes. When the cathode is over the right fronto-orbital and paired with the anode electrode over the parieto-temporal area, the two electrodes are effectively at two ends of a hypotenuse, which ensures the electricity current will pass through multiple brain areas on its way from the anode electrode to the cathode electrode, and possibly excite these areas to some degree as well. The subsequent relevant question is whether brain areas near the cathode electrode were excited or inhibited. The cathode is presumed to have an inhibiting effect (Nitsche et al., 2008), yet some studies have suggested that both the anode and the cathode have an excitatory effect on surrounding brain areas when the intensity is at 2 mA, as done in this study, rather than 1 mA (Jaconson et al., 2012). Therefore, it is possible that brain areas around the cathode were more excited than inhibited, leading to stronger results for the parietal-temporal montage. Crucially, the results demonstrate that relevant positions of the anode and cathode electrodes can impact the results found. In other words, the results found for the parieto-temporal montage, here and previously (Roncero et al., 2017), were related to the placement of the electrodes rather than just the administration of tDCS.

Comparing the different PPA sub-types, we focused on the difference found for untrained items at the final stimulation session when the parieto-temporal montage was given. This montage was chosen because it had demonstrated a significant result for untrained items, and this session was chosen because it was the least affected by post-stimulation time. While all nfPPA participants, and three of four logoPPA participants, demonstrated an improvement, only one of four svPPA participants improved. These results may be related to the principles of tDCS, which is assumed to lower the resting threshold of neurons, which in turn allows them to respond more efficiently (Nitsche and Paulus, 2000). Many neurons with borderline function in individuals with NDD might benefit from such an effect; but at the same time, there may be neurons too far from the necessary threshold needed to be affected by tDCS. For this reason, one would also anticipate that tDCS stimulation in NDD would be less effective or even ineffective in severe cases as a greater number of neurons fail to reach the threshold needed for amenability. Consistent with this argument are the results for participants with semantic variant PPA showed little improvement for untrained items, which could conceivably reflect differential effects of pathology (TDP-43 proteinopathy in semantic variant PPA vs. AD pathology in logopenic PPA); however, we would argue it more likely reflects greater loss of semantic storage of items in semantic variant PPA, reflecting the importance of a semantic hub for lexical recall in the ATL (Visser et al., 2010). Degeneration of a semantic hub, a lexicon where labels are presumably stored, produces a state where semantic variant PPA participants are unable to recall the names of objects because those names are effectively absent from the lexicon. If such labels are literally absent from semantic memory, they are unlikely to be recalled, nor benefit from stimulation, unless explicitly taught to the participant.

### Comparison to Past Studies and Implications for Future tDCS Studies Regarding Montage Choice

Despite the small sample size, the results do replicate results found in previous studies. The results found for the DLPFC montage, for example, reproduce results found by Cotelli et al. (2014), who also placed the anode electrode over the DLPFC area and the cathode over an extra-cephalic area. For trained items (which they call treated), there was an improvement for both anodal and placebo (i.e., sham stimulation), but improvement was larger for anodal stimulation. In contrast, scores for untrained items were similar regardless the stimulus condition and remained relatively unchanged. In the present study, the DLPFC montage also failed to produce an improvement for untrained items, whereas an improvement was observed for trained items when the DLPFC montage was given, which was larger than that observed for the SHAM montage. The results are also comparable to those found by Tsapkini et al. (2018) who compared PPA subtypes with anodal stimulation to the left inferior frontal gyrus and the cathode over the right cheek. As done in this study, there were a set of trained and untrained items, and while all PPA groups improved for trained items, there was a lack of improvement for untrained items in the svPPA group, similar to this study. Because the inferior frontal gyrus and the inferior parietal-temporal region are intricately connected via the articulate fasciculus for language (Catani et al., 2003), it is possible that both studies found similar results from effectively stimulating the same neural substrate. Alternatively, as previously mentioned, the similar results found for svPPA participants could reflect the degradation of a semantic hub.

### Limitations

Despite screening many patients, and enrolling a larger number of participants, only a small group of participants completed three rounds of stimulation without suffering a severe progression decline. Six months can be a long time for someone with a neurodegenerative disease, such that additional rounds and increased time run the risk of increased attrition. In the present study, for example, 27 participants completed their first round, and 23 participants completed the second round. The greatest impact on attrition was the third round (i.e., the final 2 months). Six participants withdrew after round 2, leaving 17 to complete round 3, but four of these participants had disease declines to the extent where they could no longer properly follow the study protocol, and one participant was re-diagnosed as non-PPA, leaving useable data for only 12 participants. In summary, adding branches to studies to explore additional variables has clear advantages, but attrition will be progressively worse as the chance of withdrawal and disease progression increases with each interval added. Also, due to safety concerns, trainers were left unblind, which may have influenced the results observed for trained items despite always following the same protocol. At the same time, we note that the results found for untrained items are less easily explained as these items were never trained. Similarly, while trainers may have influenced the results by unknowingly behaving differently when tDCS was real or sham, the different results found for the two real montages (DLPFC, parito-temporal) are less easily explained, especially as they produced similar results at the end of training, with differences emerging post-stimulation. We also perceived mood improvements in a few participants, but no measures of mood were administered. At the same time, it can be difficult to determine if such mood changes were due to tDCS or from simply participating in the study.

### Conclusion

Neuromodulation as a therapy for brain disease is in its early stages of development. There are numerous questions to be addressed, including: optimum dose, duration of intervention, location (of both anode and cathode), technique, and which patient populations will benefit. Some have proposed that since brain networks are the critical substrate for cognition, it matters little where the stimulation is administered. Our results suggest otherwise – these variables are likely to be critical for success, and extensive work will need to be done to evaluate whether the technique of tDCS has potential as an ancillary therapy for neurodegenerative diseases.

Negative studies are therefore to be expected as the critical variables are worked out. Nevertheless, the demonstration of significant and long-lasting benefit even in this small group of individuals suggests that tDCS should be further investigated for its practical potential as therapy in neurodegenerative diseases.

## Data Availability Statement

The datasets generated for this study are available on request to the corresponding author.

## Ethics Statement

This study was carried out in accordance with the recommendations of the internal research board of the Jewish General Hospital with written informed consent form all subjects. All subjects gave written informed consent in accordance with the Declaration of Helsinki. The protocol was approved by the internal research board of the Jewish General Hospital.

## Author Contributions

CR contributed to the study design and conceptualization, analysis of data, and drafting the manuscript. ES, MD, and SP analyzed and interpreted the data. AP drafted the manuscript. AT contributed to the study design and conceptualization. HC contributed to the study design and conceptualization, and drafting the manuscript.

## Conflict of Interest

The authors declare that the research was conducted in the absence of any commercial or financial relationships that could be construed as a potential conflict of interest.

## Footnotes

1 www.neurobs.com
